# Experiences of participants of a volunteer-supported walking intervention to improve physical function of nursing home residents – a mixed methods sub-study of the POWER-project

**DOI:** 10.1186/s12877-023-04044-4

**Published:** 2023-06-01

**Authors:** Sabine Weissbach, Anja Rieckert, Christine Kersting, Nina Grede, Norbert Donner-Banzhoff, Andreas Soennichsen, Horst Christian Vollmar, Ina Otte, Pia Weimer, Ulrike Sonja Trampisch

**Affiliations:** 1 Institute of General Practice and Family Medicine, Witten/Herdecke University, Alfred- Herrhausen-Strasse 50, 58448 Witten, Germany; 2 Department of General Practice/Family Medicine, Philipps-University Marburg, Marburg, Germany; 3 Department of Family Medicine, Ruhr University Bochum Medical School, 44801 Bochum, Germany; 4 Department of Geriatric Medicine, Marien Hospital Herne, Ruhr University Bochum, Hölkeskampring 40, 44625 Herne, Germany; 5 Research Initiative Health for Austria, Wissenschaftliche Initiative Gesundheit für Österreich, Vienna, Austria

**Keywords:** Physical activity, Quality of life, Nursing home, Oldest old, Volunteer work, Barriers, Motivators, Implementation

## Abstract

**Background:**

Regular physical activity improves physical health and mental well-being and reduces the risk of falling in older adults. The randomized controlled “**P**revention by lay-assisted **O**utdoor-**W**alking in the **E**lderly at **R**isk” POWER-study investigates whether volunteer-supported outdoor-walking improves physical function and quality of life in older people living independently or in nursing homes. This sub-study explores the experiences of older participants and volunteers in relation to their physical and psychosocial well-being as well as the challenges faced by both groups. A further aim was to explore volunteers’ experience with people living in nursing homes during the first pandemic lockdown (spring 2020).

**Methods:**

The sub-study was designed as mixed-methods approach consisting of 11 individual semi-structured guide-based interviews (nursing home residents), two focus group interviews (volunteers), and a cross-sectional questionnaire survey (volunteers). The interviews were audiotaped, transcribed verbatim, and analyzed by content analysis as described by Kuckartz. Topics addressed in the interviews were triangulated by means of a questionnaire. The quantitative data were analyzed using descriptive statistics.

**Results:**

Participants’ evaluation of the intervention was generally positive. Nursing home residents appreciated the social interaction associated with the assisted walking, which motivated them to take part regularly, provided a sense of safety, and caused pleasure on both sides. The impact on physical health status of the nursing home residents of this sub-study varied to a large degree as reported in interviews: in some cases, an improvement in physical performance, a decrease in physical complaints, and an improvement in gait or independence was reported. If not, reference was made to previous or sudden illnesses and the advanced age of the participants. Despite the COVID-19-lockdown and the associated restrictions, about 60% of contacts were still possible and participants planned to continue the assisted walks after the lockdown.

**Conclusion:**

Volunteers have a positive effect on the quality of life, mobility, and general health of nursing home residents. Even more than the improvement of physical performance, social interaction was seen as helpful. Despite their advanced age, the nursing home residents were curious and open to new contacts. When removing the identified barriers, it might be possible to integrate this program into the long-term everyday life of nursing homes.

**Trial registration:**

DRKS-ID: DRKS00015188, date of registration: 31.08.2018.

**Supplementary Information:**

The online version contains supplementary material available at 10.1186/s12877-023-04044-4.

## Background

In Germany, the proportion of people aged 65 years and older increases: while the Federal Statistical Office reports about 20% in 2019 [1], the proportion is estimated to increase to 26% by 2040 [2]. At the same time, the number of care-dependent elderly, such as nursing home residents (NHR), is expected to increase to about 50%. For comparison: In 2019, 818,317 persons in Germany needed long-term care [3]. Of those, about 20% were cared for in nursing homes with 92.6% of them being ≥ 65 years of age [4]. At this age, the risk of suffering from chronic disease, metabolic conditions, and mental disorders as well as the risk of falls rapidly increases [5]. In addition, a German population-based study showed that the risk for mental disorders (e.g., depression) increased with the number of chronic diseases [6]. To prevent the development or exacerbation of chronic diseases, mental disorders, and falls in the elderly, regular physical activity is essential [7]. The benefits of regular physical activity are higher self-esteem, a better quality of life, and fewer physical limitations in the very elderly [8]. Additionally, physical activity can reduce symptoms of depression [9].

Several studies evaluated intensive multidisciplinary programs for elderly that require substantial resources and are difficult to implement on a population basis. Other programs required costly health professionals, e.g., physiotherapists or nurses for an exercise program on an individual basis. Interventions were complex and required professional input [10, 11]. In the randomized controlled intervention study (RCT) “Home-Based Exercise Supported by General Practitioner Practices” (HOMEfit), strength, balance and mobility exercises were taught in the GPs practice to chronically ill and mobility-impaired elders through a cooperation between family physicians and exercise therapists. The actual benefits for participants living independently at home were rated as rather low [12]. Studies in which physical activity interventions for seniors are supported by volunteers target individuals living independently in the community, but never multimorbid, very frail, and mobility-impaired seniors living in nursing homes [13]. It remains unclear whether a low-threshold simple intervention such as regular walks can achieve better or equal results in this group. To clarify this question, the simple intervention of POWER (Prevention by lay-assisted Outdoor-Walking in the Elderly at Risk) has been conceptualized and piloted [14]. This intervention of a volunteer-supported outdoor-walking is targeted at older people with inadequate activity levels, already present minor restrictions in physical and mental functioning preventing them from self-paced physical activity, and a lack of support. Alternatively (e.g., in bad weather), a balance and strength exercise program from the federal center for health education was provided [15]. This 6-month intervention was tested in a randomized controlled trial for its effectiveness in improving physical performance and quality of life from January 2019 to January 2021 in Marburg/Hesse and Witten/North Rhine-Westphalia (Germany) [16].

The main study addresses older people regardless of their living situation; participants were community dwelling (Marburg/Hesse) or living in nursing homes (Witten/North Rhine-Westphalia). However, elderly living in nursing homes are more often characterized by multi-morbidity and frailty, and are barely able to independently cope with activities of daily living compared to community dwelling older adults [5, 17]. Furthermore, this group of people is often socially isolated, even though living in the community of the nursing home [18]. This sub-study explores the experiences of nursing home residents and supportive volunteers with a physical activity intervention as well as the facilitators and barriers to integrating the intervention into existing structures and processes as perceived by both groups. In addition, the sub-study evaluated challenges associated with the lockdown due to the COVID-19-pandemic in spring 2020 from the volunteers’ perspective as part of the intervention period concurred with the lockdown.

This manuscript solely reports on the sub-study. The results of the main study will be available and reported in 2023.

## Methods

The sub-study was designed as sequential mixed-methods study equally involving (1) qualitative and (2) quantitative methods [19, 20]:

1. Qualitative study: To explore the experiences of participating NHR as well as volunteers and to identify facilitators and barriers for implementing the intervention, individual semi-structured interviews were conducted with NHR, while volunteers were interviewed in semi-structured homogenous focus group interviews. For the NHRs the individual approach was used as discussions in a group environment are often difficult due to physical limitations of the elderly. Semi-structured interviews were used as they allow in-depth exploration of participants’ responses [21].
2. Quantitative study: To triangulate the results of the findings derived from the focus groups with the volunteers in a larger sample and to avoid selection bias due to possible overrepresentation of highly motivated volunteers, an additional cross-sectional questionnaire survey was carried out among the volunteers. It also addressed the volunteers’ experiences regarding contacts to participating NHR during the first COVID-19-lockdown between March and June 2020 [22].

The qualitative study reports data according to the Consolidated Criteria for Reporting Qualitative Research (COREQ) guidelines [23], whereas the cross-sectional study is reported according to the STROBE statement checklist for cross-sectional studies [24].

### Participants

This sub-study solely reports data collected from NHRs and volunteers enrolled in the POWER-study by the study center at Witten/Herdecke University. This is reasonable as the sub-study focusses on participants living in nursing homes, who were only recruited by Witten/Herdecke University. All NHRs and volunteers did not know each other before the intervention started.

### Recruitment

1. Qualitative study:

Individual interviews: At least 10 interviews were planned. The number of available NHRs was limited. Twenty-two of 60 NHRs enrolled in the POWER-study (study center Witten/Herdecke University) and receiving the intervention were eligible for the interviews as they completed follow-up data collection for the main study, were in good state of health, able to communicate and had sufficient cognitive capacity (according to the subjective assessment of nursing home staff). Of those, eleven participants were randomly invited in a first wave by letter to participate in a face-to-face interview. All of them agreed.

Focus groups: Two focus groups with four to eight participants were planned to limit bias. In total, 73 volunteers were trained by the study center at Witten/Herdecke University prior to the start of the intervention and were closely supported by the study center during the intervention. Of those, 50 performed assisted walks during the study. They were assigned to the participating NHRs by chance. Volunteers could choose three nursing homes from a list of nursing homes that were accessible to them. Volunteers who regularly carried out visits or assisted walks and whose assigned NHR had completed the follow-up assessment for the main study, were eligible. Using a random order list, twenty volunteers fulfilling these criteria were invited by letter. Twelve finally participated.

Participation in the qualitative study was voluntary. All interview and focus group participants received an expense allowance (30 €). Participants provided informed, written consent before participation.

(2) Quantitative study:

All volunteers enrolled to the study via Witten/Herdecke University and who performed more than one visit in nursing homes were invited to participate in the survey (n = 50). The paper-based questionnaire was administered via post in June 2020. At this time, follow-up data collection for the main study was completed for all participating NHRs and assisting volunteers. To achieve a high response, up to two reminders were sent by email and post between July and August 2020. A last reminder call was made in September 2020. Participation was voluntary. Participants did not receive any incentive.

### Data collection

1. Qualitative data:

Interviews and focus groups were conducted to elaborate on the NHRs’ and volunteers’ motivation for participation, self-perceived physical and mental effects of intervention, additional experiences of intervention, perceived challenges, project organization, and continuation/implementation into daily practice routine in the life of the elderly, nursing homes and volunteer organizations. The interview guide was identical for individual interviews and focus groups. The concept of information power was followed.

Interviews were carried out until theoretical saturation to the aim and analysis of the study as well as to sample specificity and the generated quality of dialogue. No new themes were identified after 7 interviews and no new themes arose in the focus groups who were timely held after the interviews [25]. Interviews and focus groups were conducted in November and December 2020.

Individual interviews: Face-to-face interviews were conducted at the participants’ room or apartment in the nursing home. The interviews lasted between 9 and 25 min.

Focus groups: Two focus groups (the first with 8 participants, the second with 4 participants) were undertaken in November and December 2020 to explore volunteers’ experience with the intervention. Focus groups were carried out in the rooms of Witten/Herdecke University and lasted one hour each.

The interview guide (Supplement 1) for the individual interviews as well as the focus group interviews was developed by a medical student (PW), who was supervised by a research assistant qualified and well experienced in qualitative research (AR). PW conducted all individual interviews, and both focus group sessions. She was not involved in the POWER-study otherwise and had not met any of the participants before. A study assistant experienced in qualitative research (SW) organized the focus group interviews and took notes during the focus groups, a research assistant (Doctor of Sport Science) moderated the discussion (UT). At the start of each interview and focus group, it was made clear that all members of the study personnel were independent and that opinions in all directions could be expressed without any consequences. All interviews were audio-recorded and transcribed verbatim.

(2) Quantitative data:

An eight-page written questionnaire (Supplement 3) for volunteers was developed, based on the interview guide and the results of the focus group interviews. In detail, it enquired about motives for participation, training and support, information about progress of intervention, personal physical and mental experiences, requirements for further volunteer work and implementation of the project in existing structures. The COVID-19-pandemic that occurred in spring 2020 raised additional questions that concerned contacts with participating NHRs during the lockdown.

Attitudes were assessed with a 5-point Likert-scale, but respondents also had the option to answer, “don’t know.” A free text field was offered for additional comments.

### Data management and analysis

1. Qualitative data:

To facilitate analysis, all audio-recordings were transcribed verbatim, anonymized, and imported to MAXQDA, Version 20.3. For analysis, all interviews were considered at full length. Thematic qualitative content analysis with a deductive-inductive approach was performed to analyze the material as described by Kuckartz [26]. The data were analyzed by three researchers (PW, UT and SW), who independently coded the material in a 7-step approach: First, all coders independently read all transcripts and initially worked with the text by highlighting important passages and composing memos. Second, main topical categories were developed from the interview guide, and third, the first coding process was conducted by coding the entire material using the main categories. Fourth, all passages were compiled and assigned to each of the main categories, and fifth, subcategories were determined inductively from the material. The sixth step was the second coding process: coding all the data using the elaborate system. Thereafter, as a seventh step, a category-based analysis along the main topics was conducted. Any disagreements were solved by discussion. Contradictory data were considered. The final coding scheme is presented in Supplement 2.

(2) Quantitative data:

To analyze the data assessed via questionnaire, descriptive statistics were performed using IBM SPSS Statistics for Windows, Version 27.0 (Armonk, NY: IBM Corp.). Means and standard deviations (SDs) were used for continuous data. Categorical variables are shown as n (%).

Data of the (1) qualitative and (2) quantitative analysis were merged by constructing a joint display, which helped to get a comprehensive overview and to discuss the experiences of both NHRs and volunteers.

## Results

Results are presented by topic (1 = results of the qualitative study, 2 = results of the quantitative data). Additional files show results of qualitative and quantitative analysis in more detail (see additional files 1–6).

### Characteristics / study participants

1. Qualitative study:

Overall, eleven NHRs and twelve volunteers took part in the qualitative study. The NHRs were between 74 and 95 years old, nine of them (81.8%) were female. The volunteers (n = 12) were aged 39 to 73 with most of them being female as well (66.7%).

The six main categories for analyzing the interview material deductively emerged from the interview guide were the following: Motivation for participation, self-perceived physical and mental effects of the intervention, additional experiences during the intervention, perceived challenges, project organization, and continuation/implementation. Within these main categories, twenty subcategories were identified inductively (see supplement 2).

(2) Quantitative study:

In total, 40 of 50 questionnaires were returned by volunteers (response rate: 80%). The participants were aged 18 to 84 years old (mean 56 ± 18.2 (SD)), 30 of them were female (75%) (Table 1). Depending on time availability of the individual volunteer, NHRs could be accompanied by up to 3 volunteers to cover the required number of walks per week. Volunteers were welcome to accompany more than one NHR (in the same or in different care homes) to ensure that the intervention could be realized for all participants of the intervention group (number of NHRs accompanied n = 54).

**Table 1:** Characteristics of interview participants and volunteers values are numbers (percentages) unless stated otherwise

	Nursing home residents	Volunteers	
	Interviews (N = 11)	Questionnaire (N = 40)	Focus groups (N = 12)
**Age** mean ± SD (range)	85.8 ± 6.8 (74–95)	56 ± 18.2 (18–84)	64 ± 9.1 (39–73)
**Women**	9 (81.8)	30 (75.0)	8 (66.7)
**Marital status**			
married	0 (0)	18 (45.0)	7 (58.3)
single	0 (0)	15 (37.5)	3 (25.0)
divorced	0 (0)	5 (12.5)	2 (16.7)
widowed	11 (100.0)	2 (5.0)	0 (0)
missing	0 (0)	0 (0)	0 (0)
Education level*			
low	4 (36.4)	9 (22.5)	2 (16.3)
medium	6 (54.5)	10 (25.0)	1 (8.3)
high	1 (9.1)	21 (52.5)	9 (75.0)
**Born in**			
Germany	11 (100.0)	34 (85.0)	10 (83.3)
other	0 (0)	1 (2.5)	0 (0)
missing	0 (0)	5 (12.5)	2 (16.7)
**Further voluntary engagement**	n/a	21 (52.5)	5 (41.7)
**BMI** mean ± SD (range)	27.1 ± 5.2 (20.3–36.4)	n/a	n/a
**Care degree**			
1	0 (0)	n/a	n/a
2	8 (72.7)	n/a	n/a
3	2 (18.2)	n/a	n/a
4	1 (9.1)	n/a	n/a
5	0 (0)	n/a	n/a
**Use of walking aid**			
none	3 (27.3)	n/a	n/a
cane	0 (0)	n/a	n/a
walker	8 (72.7 )	n/a	n/a
**MMSE** (Mini mental status examination) – baseline only mean ± SD (range)	26.1 ± 3.1 (21–30)	n/a	n/a
**SPPB** (Short physical performance battery) mean ± SD (range)	4.4 ± 1.3 (2–6)	n/a	n/a
**EQ-5D-5 L** (Quality of life) mean ± SD (range)	0.7 ± 0.2 (0.4 − 1)	n/a	n/a
**Clinical frailty scale** ^**†**^			
Very fit	0 (0)	n/a	n/a
Well	0 (0)	n/a	n/a
Managing well	1 (9.1)	n/a	n/a
Vulnerable	2 (18.2)	n/a	n/a
Mildly frail	3 (27.3)	n/a	n/a
Moderately frail	3 (27.3)	n/a	n/a
Severely frail	2 (18.2)	n/a	n/a
Very severely frail	0 (0)	n/a	n/a
Terminally ill	0 (0)	n/a	n/a

*According to the international standard classification of education 1997 (ISCED-97)

^†^ Rockwood et al. 2005

Demographic and clinical characteristics of the NHRs and volunteers are shown in Table 1.

### Motivation for project participation

1. Main motivation for participating in the project was the interest in physical activity for both sides. NHRs expected an improvement in physical fitness and gait as well as an opportunity to “get out*”. “Well, how can I say it? I always wanted to learn to walk again. I almost couldn’t walk at all anymore.”* (NHR-W4).

Besides personal fulfilment, many volunteers were also interested in taking part in the scientific study.

(2) Based on the survey data the most important reasons of volunteers were (multiple answers possible): interest in physical activity (n = 21; 52.5%), gaining experience (n = 20; 50.0%), interest in scientific study (n = 17; 42.5%), giving meaning to my life/altruism (n = 16; 40.0%) and new contacts (n = 14; 35.0%).

More information is provided in additional file 1.

### Self-perceived physical and mental effects of intervention

1. Consistently, the effects on the physical well-being of the NHR were reported by both NHR and volunteers to vary widely: In some cases, improvement in physical performance, increase in the length of walks, regaining of skills such as independent stair climbing, coping with inclines, and decrease in physical complaints such as shortness of breath were observed: The companionship of the helpers extended the radius of the walks and activities:

*“Yeah, that just got me out. Because I / I wasn’t even in the garden yet. And then I also went around the square once with Ms XX and I saw completely strange streets or things / so I also crossed the street sometimes” (NHR-W1).*

But even without companionship the self-confidence grew and the fear of falling decreased:

*“Imagine all the things you couldn’t do in the last few months. You’ve learned all that now. And that’s how I see it, too. That I can move around quite freely now and only through these walks back and forth” (NHR-W10).*

The NHRs enjoyed increasing independence, but sometimes also found the program stressful and were glad to see the end of the observation period. Volunteers referred to pre-existing conditions independent from the study, acute illnesses, and advanced age of the NHRs that negatively affected overall physical performance and in some cases led to discontinuation of the walks: *“…sometimes it is progress when there is no step backwards“ (V-F2B4).*

Regarding mental well-being, the importance of conversations was unanimously stressed: “*We laugh a lot. Moreover, it gives me other ideas. Not always just sad ones. When you’re in a situation like that, you’re always in the mood to complain.” (NHR-W4)*.

NHRs experienced new courage to face life and distraction from negative thoughts, but in some cases also felt burdened by the volunteers’ private problems or stressed by the physical strain and commitment of the intervention. The volunteers found the regular walks relaxing and used the opportunity to increase their own physical activities (e.g. getting there by bike or subsequent Nordic walking training). The insight into the life of the NHR, the joy and gratitude of the seniors and the intergenerational exchange were seen as extremely positive and important for their own development: *“Then I always think to myself, I’m still a young person and I should actually change that now [to avoid the poor living conditions in the nursing home later on in my life]. I have to take responsibility now, so that when I’m that age, the change is there. That I don’t have the same problem later on.” (V-F2B5)*.

(2) Descriptive evaluation of the questionnaire showed a slight self-perceived improvement in physical health of volunteers (n = 32; 80% no difference; n = 8; 20% improved/more likely improved), but no increased physical or psychological stress due to the intervention (n = 39; 97.5% reported no or more likely no physical overload; n = 38; 95% reported no or more likely no psychological overload).

For more details see additional file 2.

### Additional experiences of the intervention

1. Almost without exception, a good to very good relationship was reported between NHR and the accompanying volunteer, despite the random assignment of the walking partners: *“Ms XX, that could be my sister” (NHR-W9)*.

In some cases, a relationship of trust developed that led, for example, to the issuing of powers of attorney for the volunteer: *“She said: The good Lord sent you” (…) I even have power of attorney. So that means something, doesn’t it? (…) When you have such confidence that you are allowed to do this and that, that is unbelievable.” (V-F1B4)*.

The volunteers repeatedly motivated the NHRs to go for walks and responded to their needs. The NHRs in turn expressed their joy and gratitude and were often persuaded to go for a walk. Both NHR and volunteer recognized that the NHR felt safer on the walks because of the accompaniment and that the fear of falling was reduced: *“Of course, she wouldn’t have done it on her own, she would have been too scared or something, but this way she had the confidence to do it voluntarily. And then on the way back she also went up the stairs again.” (V-F1B7)*.

Volunteers explained the lack of motivation of NHRs with strongly fluctuating health conditions: *“The motivation was always very different. It is always according to the personal situation. Whether the lady feels good that day or not. Sometimes there was also a day when I thought, why doesn’t she want to go out now?” (V-F2B5)*.

Some NHRs were not always clear about the extent of the intervention when agreeing to participate. Therefore, in rare cases, there was a refusal or denial of the walks and exercise opportunities. The BZgA’s exercise booklet, which was intended as an alternative to the walk, was used rather rarely. Instead, both the NHR and the volunteers preferred walking in the long corridors of the nursing homes when outdoor walks were not possible (additional file 2).

(2) 47 of all participants (87%) described their relationship to the NHR as good or very good. 75% (n = 40) even reported that they developed a trustful relationship. Only few described the relationship as bad (n = 1, 1.9%) or without trust (n = 3, 5.6%). For more information see additional file 3.

### Perceived challenges

1. Besides the task of motivating the NHR to go for a walk, the responsibility during the walks as well as unexpected events occurring on the way were also a challenge for the volunteers. The proximity and contact to the nursing home gave the volunteers safety. Sudden events during the walks, such as disorientation or physical discomfort, were rarely reported: *“I was (…) was pretty much at the beginning, still a stranger, not doing anything big (…) and she couldn’t walk any further. Oh, I ran to bring the wheelchair down. When she was sitting in it, I was happy. I brought her up, water, water, water, and then I went to the nursing staff and told them, but as I said: in retrospect it was nothing bad, but in the situation: no, it wasn’t great.” (V-F1B2)*.

Although these events unsettled the volunteers, they were no reason for permanent discontinuation of walks. A lack of information about the intervention (e.g. due to cognitive limitations) required significant efforts to motivate NHRs and in some cases led to the end of the physical activity: *“The second lady didn’t really know herself what she was getting into, I think. And after a short time she said “no, I don’t need to take part in any more Olympics”.” (V-F1B2)* Implementation of walks was sometimes made more difficult by the environment of the nursing home, which was not suitable for walks, or by weather conditions such as high temperatures or rain.

(2) The main reason for cancelling walking appointments by the NHR (or the nursing home staff) was poor health (n = 24; 44.4%) followed by lack of motivation (n = 14; 25.9%) of the NHR. 25.9% (n = 14) of the responding volunteers had no dates cancelled by the NHR. Reasons for permanent cancellation of the intervention by the NHRs were lack of motivation (n = 10; 18.5%), bad health conditions (n = 2; 3.7%) or in 29.6% (n = 16) other reasons (e.g. end of observational period (multiple answers possible).

For more details see additional file 4.

### Project organization

1. The random assignment of volunteers and NHRs of different ages and genders was unanimously described as stimulating: *“It is not a question of age “(NHR-W8)*.

The volunteers recognized that the walks would not continue independently without constant encouragement from outside. Therefore, they suggested the involvement of family members or the formation of teams among the NHRs for mutual motivation: *“…and I can’t imagine that (…) it will be continued independently on its own. It quickly goes into the old rut, if one had perhaps forged alliances, in pairs, small groups, not groups, two, then perhaps one would have pulled the other along” (V-F1B2)*.

Preference should be given to single and socially isolated people (judged by caregivers) in programs such as POWER: *“If it was someone who was totally single and had no one to go with them, then it would make sense to accompany them.” (V-F1B7)*.

NHRs also recommended the program for this group of people and encouraged the participation of younger and healthier people: *“Those who are younger than me would perhaps do better (…). But I’m telling you, most of the people who are here are all in wheelchairs “(NHR-W1)* They expected volunteers to be reliable and to be able to motivate.

2. Respondents rated the following aspects as important for further voluntary work (multiple answers possible): the accessibility of place of work (n = 33; 84.6%), time flexibility (n = 29; 74.4%), insurance (n = 23; 59.0%), and professional support (n = 21; 53.8%). Only a minority considered a financial compensation as an important factor (n = 2; 5.1%). 70% of the respondents (n = 28) can imagine further volunteering.

Additional file 5 shows that in more detail.

### Continuation and implementation

(1)Generally, NHRs and volunteers enjoyed the project and would like to stay in touch: *“We have agreed that we will continue to walk together. Twice a week.” (V-F2B3)* The NHRs’ focus tends to be more on social contact and conversation than on physical activity: *“We sit outside on the bench and then talk. But outside.” (NHR-W2)* A regular continuation of the walks is seldom planned by either side and is attributed to a decline in motivation for physical activity by the volunteers *“I was glad when that was over. Because then I was no longer able to cope so well. And so I was glad when it was over.” (NHR-W7*) or due to increasing health problems of the NHRs: *“Yes, in my case it simply ended due to illness. She was a lady with Parkinson’s disease and afterwards she was no longer allowed to walk like that and sometimes even bedridden.” (V-F1B5)*.

(2)Twentythree responding volunteers (42.6%) were still in contact with the NHRs after the end of the observation period and continued visits (n = 10, 26.3%), walks (n = 7, 18.4%), or other forms of contact like for example telephone calls (n = 6, 15.8%). Twelve of these volunteers even kept in touch in many different ways during the pandemic lockdown (e.g. telephone calls n = 7; 36.8%, correspondence n = 3; 15.8%) (multiple answers possible).

Fifteen volunteers planned to continue the visits/walks after the end of the lockdown (yes/more-likely yes 65.2%). More information is provided in an additional file (see additional file 1).

## Discussion

This analysis reports on the experiences of NHRs and volunteers who participated in a study to improve the physical performance and quality of life of NHRs. Currently, there is no related study known examining how a low-threshold physical activity (walking) intervention in nursing homes affects participant groups (multimorbid and frailty NHR and volunteers, who support them) in terms of motivation, experiences, and challenges.

Main motivation for study participation on both sides was interest in exercise, but it was challenging to implement the intervention on a regular and sustained basis. The experiences regarding social contacts between completely unknown people were overwhelmingly positive. Both groups of participants benefited from social contacts while improving physical performance in the vulnerable and multimorbid population in a nursing home did not seem to be consistently achievable.

Similar motives were found in the British ACE study, in which volunteer elderly persons assisted other seniors who are still able to live independently at home in activities outside the home environment [27]. The motivation for participation among volunteers differed slightly: while in ACE it was more personal benefits, (“having something to do”, avoiding loneliness, the need to feel needed, pleasure), in POWER the motives “gaining experience” as well as “interest in participating in a scientific study” were mentioned by the participants.

The effect on the physical performance of the NHRs varied greatly: in some cases, there were significant improvements, the volunteer helpers provided security during the walks. In other cases, the general decline in health, lack of personal initiative and motivation of the residents led to interruption or discontinuation of the physical activity intervention. While an intensive intervention like in the HOMEfit study may be rather counterproductive, such a low-threshold intervention seems to be promising [12].

The exercise recommendation of the WHO was not always achievable and seems to depend significantly on the health status of the multimorbid very old residents. These experiences are consistent with the results of other studies: in a systematic review of barriers and factors to physical activity in the very elderly (setting: community-dwelling, independent living, nursing home), the most important barriers were health and physical impairments [28]. But no studies could be identified that included only persons > 79 years of age. Therefore, identification of typical motivators and barriers for this age group was not possible [28]. Mauer et al. concluded in a systematic review identifying attitudes and needs of nursing home residents regarding physical activity that an individualized program was preferred by NHRs. Physical activity should be adapted to individual physical impairments. Information deficits, triggered by ignorance and perception disorders, can be a barrier to participation in activities offered by the nursing home [29].

Social relationships were very important to both NHRs and the volunteer helpers and seem to be the greater benefit of the study. Despite the random assignment of the NHR and volunteer tandems, relationships often developed beyond the pandemic lockdown and the study. NHRs reduced their fear of falls through companionship. Patients who have already fallen (like most NHRs) are at particularly high risk of falling again. In most cases, fear of falling is even a cause of additional falls, as it leads to unfavourable changes in gait and a reduction of mobility and thus of physical abilities [30]. Whether accompanied walks generally have a positive influence on the fear of falling - even in the case of unaccompanied walks - should be systematically assessed and evaluated in follow-up studies. Volunteers gained enriching insights into the living situation in old age. Views of older adults and awareness of the issues facing this population, including isolation, falls, and the spectrum of health conditions, are changing [31]. Working with seniors gave the volunteers satisfaction and represented for them a personal value of giving something back to society. The voluntary work helped to establish new contacts, which in turn were mostly seen as enriching for both sides and improved the quality of life. The accompaniment by the initial strangers was seen positively and quickly turned into friendly feelings and familiarity on both sides, in some cases also to an intensive relationship of trust [32]. Contact was often maintained, even during periods of visitation restriction due to the pandemic lockdown (e.g., balcony-talks), and a desire was expressed to keep contact permanently by the volunteers. On the other hand, it became apparent that older people in the nursing home are quite self-determined, this was shown in the few cases where the intervention was terminated by the NHR, despite topic-related training and support of the volunteer helpers by the study center. Some participants in the intervention were not sufficiently informed about the intervention or increasing cognitive impairment hindered implementation.

The very close relationship between the NHR and volunteers could be both, a success, and a challenge: whether a sudden break-off of contact could lead to negative feelings or psychological distress of NHRs or volunteers should be further researched in similar studies.

## Limitations

Certain limitations ought to be acknowledged: participants were recruited independently by the nursing home staff, observing the inclusion and exclusion criteria. Thus, the recruitment could have been carried out rather subjectively and “sympathetic” residents or residents interested in exercise could have been approached, which could lead to a bias in the results. Among the residents of a nursing home, the seniors interviewed belonged to a particularly severely frail group, which might have influenced the motivation for physical activity. In German nursing homes, various activities are offered, including physical activities such as seated gymnastics or short walks in groups. A review of nursing home physical activity policies or routines and NHR participation in these activities were not recorded and may have had an impact on nursing home residents’ motivation to walk with volunteers three times a week. Recalling the intervention was a problem for some interview participants due to increasing cognitive impairment and led to some very short interviews. Only a random sample of the volunteers took part in the focus groups: A possible positive selection of focus group participants cannot be excluded, and anonymity is generally lacking in focus groups. Focus group interviews did not report on any negative experiences and problems encountered during the project. A kind of group dynamic can arise in focus groups and influence statements positively or negatively. This tendency towards social desirability may have resulted in predominantly positive statements regarding the project in these focus groups. It is known that not all people want to express a critical opinion in a group: to create a more anonymous response situation, a questionnaire for all participating volunteers was developed, based on the interview guide and statements from focus group participants. This questionnaire was not validated which should be mentioned as a possible limitation. Not all volunteers answered the questionnaire. Thus, it is conceivable that those with a good relationship with the NHR and study staff were more likely to have been reached or to have participated in focus groups and questionnaires [33]. This may have influenced the results.

## Conclusion

The findings of this project highlight the value of volunteers in outreach to mobility impaired NHRs. Nursing home residents (NHR) are a vulnerable and severely frail group with limited physical conditions and low life expectancy. The simple and low-threshold intervention of the project was predominantly evaluated positively by both NHRs and volunteers and improves the quality of life of both groups. In almost all cases, the volunteers found good access to the seniors. Even though no new insights could be provided regarding physical activity, the intervention can be considered as success for the participants in any case due to the positive experiences. Due to the different life situations of the NHRs and the volunteers and the willingness of the NHRs to be physically active depending on their daily form, the regular implementation of the intervention was not always easy. More evidence is needed to identify methods that will sustain NHRs’ motivation to be physically active over the long term. Physical activity programs should be developed that are tailored to individual physical, cognitive, and mental limitations in the very old to find an optimal approach to enhance their quality of life while maintaining and improving mobility and independence. The environment of the nursing home should be suitable for a walking intervention. Future implementers of the study should consider that volunteers who accompany nursing home residents in physical activity need preliminary training, ongoing good supervision, and a contact person from the nursing home staff. In this way, volunteers can effectively support physical activity in nursing homes.

### Electronic supplementary material

Below is the link to the electronic supplementary material.

Supplementary Material 1

Supplementary Material 2

Supplementary Material 3

Supplementary Material 4

Supplementary Material 5

Supplementary Material 6

Supplementary Material 7

Supplementary Material 8

Supplementary Material 9

## Data Availability

The datasets used and/or analysed during the current study are available from the corresponding author on reasonable request.

## List of abbreviations

GP: General Practitioner
NHR: Nursing home resident
POWER: Prevention by lay-assisted Outdoor-Walking in the Elderly at Risk
RCT: Randomized Controlled Trail
WHO: World Health Organization
