# Correction: Experiences of participants of a volunteer-supported walking intervention to improve physical function of nursing home residents – a mixed methods sub-study of the POWER-project

**DOI:** 10.1186/s12877-023-04134-3

**Published:** 2023-09-11

**Authors:** Sabine Weissbach, Anja Rieckert, Christine Kersting, Nina Grede, Norbert Donner-Banzhoff, Andreas Soennichsen, Horst Christian Vollmar, Ina Otte, Pia Weimer, Ulrike Sonja Trampisch

**Affiliations:** 1 Institute of General Practice and Family Medicine, Witten/Herdecke University, Alfred- Herrhausen- Strasse 50, 58448 Witten, Germany; 2 Department of Family Medicine, Ruhr University Bochum Medical School, 44801 Bochum, Germany; 3 Department of General Practice/Family Medicine, Philipps-University Marburg, Marburg, Germany; 4 Research Initiative Health for Austria, Wissenschaftliche Initiative Gesundheit für Österreich, Vienna, Austria; 5 Department of Geriatric Medicine, Marien Hospital Herne, Ruhr University Bochum, Hölkeskampring 40, 44625 Herne, Germany

**Correction to: BMC Geriatrics (2023) 23:343**

10.1186/s12877-023-04044-4.

After publication of this article [1], the authors reported that in this article the author name ‘Weimer’ was incorrectly written as ‘Weimar’.

The original article [1] has been corrected.
