# Supplementary material for: Experiences of participants of a volunteer-supported walking intervention to improve physical function of nursing home residents – a mixed methods sub-study of the POWER-project
Source: BMC Geriatr. 2023 Jun 1;23:343. doi: 10.1186/s12877-023-04044-4 (PMC10234228; doi:10.1186/s12877-023-04044-4)
Supplement: Supplementary file 1 — Supplementary Material 1 [file 12877_2023_4044_MOESM1_ESM.pdf]

S1 Appendix Interview Guide

Topics	Questions
General	Standard opening question:<br>What was the reason you agreed to participate in the POWER project?<br>How did you find out about the project?
Expectations prior to the project	What expectations did you have prior to the POWER project?
Experiences with training, study materials and support	Can you tell me how you experienced the preparation meetings prior to the project and the exchange meetings during the project?
Relationship to the senior (test person)	Can you tell me about the first meeting with „your“ senior?<br>What relationship developed between you during the course of the project (relationship of trust)?<br>What did you talk about during the walks?
Impact of walks to the senior(intervention)	Can you tell me about the impact of the walks on the senior? Have you noticed developments?<br>Do you think positive changes in the wellbeing of the elderly could be for reasons other than walks?
Impact of walks to the volunteer	How did you experience the walks with the senior?<br>Can you tell me about situations in which you felt unsuccessful, insecure or overwhelmed?<br>How did the regular walks affect you?
Satisfaction with the intervention	Can you tell me how satisfied you are with your voluntary work in the POWER project?<br>Would you like to change something?
Future of voluntary work in the POWER project	Would you like to continue the walks with “your“ senior?<br>How will the relationship between you and the senior develop after stopping the walks?<br>Will you keep in touch even if “your” senior can no longer go for a walk?
