# Supplementary material for: Experiences of participants of a volunteer-supported walking intervention to improve physical function of nursing home residents – a mixed methods sub-study of the POWER-project
Source: BMC Geriatr. 2023 Jun 1;23:343. doi: 10.1186/s12877-023-04044-4 (PMC10234228; doi:10.1186/s12877-023-04044-4)
Supplement: Supplementary file 2 — Supplementary Material 2 [file 12877_2023_4044_MOESM2_ESM.pdf]

S2 APPENDIX Coding scheme individual interviews/focus groups

Main category	Sub category individual interview	Sub category focus group
Reason for participation	reason for participation	reason for participation
Physical and mental effects of intervention	effects on NHRs' physical well-being<br>effects on NHRs' mental well-being	effects on NHRs physical well-being<br>effects on NHRs' mental well-being<br>effects on Vs' physical well-being<br>effects on Vs' mental well-being
additional experiences of intervention	relationship of walking partners<br>behavior of V<br>alternative exercise program according to protocol<br>protection/safety for NHR on walks by V	relationship of walking partners<br>behavior of NHR<br>alternative exercise program according to protocol<br>protection/safety for NHR on walks by V
Challenges of intervention	uncertainity/overload<br>discontinuation of interverntion<br>challenges due to general conditions	uncertainity/overload<br>responsibility for NHR<br>challenges due to NHRs behaviour<br>challenges due to general conditions<br>discontinuation of intervention
Future/continuation	continuation of contacts planned/not planned	continuation of contacts planned/not planned
Project organization	matching NHR-V<br>selection of NHR and V/recommendations	matching NHR-V<br>selection of NHR/recommendations

NHR= participating nursing home residents, V=volunteer,
