# Supplementary material for: Experiences of participants of a volunteer-supported walking intervention to improve physical function of nursing home residents – a mixed methods sub-study of the POWER-project
Source: BMC Geriatr. 2023 Jun 1;23:343. doi: 10.1186/s12877-023-04044-4 (PMC10234228; doi:10.1186/s12877-023-04044-4)
Supplement: Supplementary file 3 — Supplementary Material 3 [file 12877_2023_4044_MOESM3_ESM.pdf]

Additional file 1. Motivation for project participation and sustainable implementation
*NHR=nursing home resident, V=Volunteer, 1=individual interview, 2=focus group, *multiple response possible, †=data given for 54 NHR*

	Qualitative study		Quantitative Study	
	**Key results**	**Example**	**Variables assessed in the questionnaire**	**Response category N (%)**
**Motivation for project participation**	<u>Consensus:</u><br>+ Interest in exercise<br><u>NHR:</u><br>+Improvement of physical fitness and gait pattern<br>+ Opportunity to "get out"<br>- Pressure due to fixed appointment<br><u>V:</u><br>+ Doing good/meaningful things (personal fulfillment)<br>+ Interest in the topic	"Well, how can I say it? I always wanted to learn to walk again. I almost couldn't walk at all anymore." (NHR-W4)<br><br>"I thought I would come as a completely new person and give a completely different input for people. That was one thing, the other was: I like to move and I thought it would be good for others too. So that's a win-win story, I thought." (V-F1B5)	Motives for participating in the project*	Interest in physical activity 21 (52.5)<br>Gaining experience 20 (50.0)<br>Interest in scientific study 17 (42.5)<br>Meaning of life 16 (40.0)<br>New contacts 14 (35.0)<br>Improving one's own health 2 (5.0)<br>Receiving recognition 2 (5.0)<br>Financial Reward 1 (2.5)<br>others 8 (20.0)<br>Don`t know 0 (0)<br>Not stated 0 (0)<br>Missing 0 (0)
**Continuation & Implementation**	<u>Consensus:</u><br>+ Keep in contact<br>+ Contact/walks rarely regular<br><u>NHR:</u><br>+ Focus on social contacts/conversation<br>- Discontinuation of contact/walks for no apparent reason for NHR<br><u>V:</u><br>- decreasing motivation of NHR for physical activity<br>- Physical activity could often not be continued due to health problems of NHRs	"We sit outside on the bench and then talk. But outside." (NHR-W2)<br>"I said get in touch. However, no one came forward. "(NHR-W5)<br><br>"We have agreed that we will continue to run together. Twice a week."(V-F2B3)<br><br>"Yes, in my case it simply ended due to illness. She was a lady with Parkinson's disease and afterwards she was no longer allowed to walk like that and sometimes even lay down." (V-F1B5)	Contact with V at the beginning of the lockdown*	Visits 10 (32.3)<br>Walks 7 (22.6)<br>Other 6 (19.4)<br>No 15 (48.4)<br>Missing 9 (22.5)
			Contact during the lockdown*	Telephone calls 7 (36.8)<br>Correspondence (letter/Whatsapp/SMS) 3 (15.8)<br>Other (shopping for PRO, talking over the balcony) 3 (15.8)<br>Walks/visits (with special permission) 2 (10.5)<br>No 7 (36.8)<br>Missing 0 (0)
			Planned restart of walks after the lockdown	Yes 11 (47.8)<br>More-likely yes 4 (17.4)<br>No 2 (8.7)<br>More likely no 0 (0)<br>Don't know 2 (8.7)<br>Not stated 0 (0)<br>Missing 4 (17.4)
