# Supplementary material for: Experiences of participants of a volunteer-supported walking intervention to improve physical function of nursing home residents – a mixed methods sub-study of the POWER-project
Source: BMC Geriatr. 2023 Jun 1;23:343. doi: 10.1186/s12877-023-04044-4 (PMC10234228; doi:10.1186/s12877-023-04044-4)
Supplement: Supplementary file 4 — Supplementary Material 4 [file 12877_2023_4044_MOESM4_ESM.pdf]

Additional file 2. Physical and mental effects on nursing home residents (NHR) and volunteers (V) due to intervention
NHR=nursing home resident, V=Volunteer, 1=individual interview, 2=focus group

	Qualitative study		Quantitative Study	
	**Key results**	**Example**	**Variables assessed in the questionnaire**	**Response category**
**Effects on NHRs' physical well-being[1,2]**	Consensus:<br>+ partial permanent increase in performance<br>+ partial decrease in physical complaints<br>- partial increase in physical complaints (due to existing underlying diseases/age of PRO).<br>NHR:<br>+ increasing independence<br>- partial relief about the end of the walks (observation period)<br>V:<br>- partial discontinuation of walks	"…sometimes it is progress when there is no step backwards" (V-F2B4)<br>"Imagine all the things you couldn't do in the last few months. You've learned all that now. And that's how I see it too. That I can move around quite freely now and only through this walks back and forth" (NHR-W10)<br>Yeah, that just got me out. Because I / I wasn't even in the garden yet. And then I also went around the square once with Ms XX and I saw completely strange streets or things / so I also crossed the street sometimes.(NHR-W1)	Frequency of walks/week	<1x/week 8 (14.8)<br>1x/week 19 (35.2<br>2x/week 24 (44.4)<br>3x/week 2 (3.7)<br>>3x/week 1 (1.9)
**Effect on NHRs' mental well-being [1,2]**	Consensus:<br>+ Participants became more open in the course of time<br>+ Social exchange/conversations important and positive<br>NHR:<br>+ new courage to face life, distraction from gloomy thoughts<br>- Fear of excessive demands and obligations<br>- unreliability and private problems of the volunteers	"We laugh a lot. Moreover, it gives me other ideas. Not always just sad ones. When you're in a situation like that, you're always in the mood to complain." (NHR-W4)<br>"Then we sat there for half an hour doing fractions. He wanted to try shorthand again. Then I asked again, I used to be able to do it, and I tried to be a bit more firm again." (V-F2B2)	n/a	**n/a**
**Impact on Vs' physical well-being[2]**	V:<br>+ walks as relaxing<br>+ increased sporting activity by cycling or Nordic walking afterwards	"...because the way with the bike is already good for me then. The rest is good for both of us" (V-F2B3)	Self-perceived improvement in V's physical performance and health	Improved 3 (7.5)<br>More likely improved 5 (12.5)<br>No difference 32 (80.0)<br>More likely impaired 0 (0)<br>Impaired 0 (0)<br>Missing 0 (0)
			V's self-perceived physical overload due to intervention	Overloaded 0 (0)<br>More likely overloaded 1 (2.5)<br>Neither nor 0 (0)<br>More likely not overloaded 4 (10.0)<br>Not overloaded 35 (87.5)<br>Missing 0 (0)

Additional file 2. Physical and mental effects on nursing home residents (NHR) and volunteers (V) due to intervention
NHR=nursing home resident, V=Volunteer, 1=individual interview, 2=focus group

	Qualitative study		Quantitative Study	
	**Key results**	**Example**	**Variables assessed in the questionnaire**	**Response category**
**Impact on Vs' mental well-being$_2$**	V:<br>+ Insight into life in the elderly<br>+ Joy and gratitude of the NHR<br>+ a meaningful task<br>+ intergenerational instructive exchange<br>- partly poor living conditions in the nursing home	"The arrival, a little bit of walking, a little bit of chatting, for the lady it was positive, for me it was positive...." (V-F1B5)<br><br>"Then I always think to myself, I'm still a young person and I should actually change that now. [the poor living conditions in the nursing home] I have to take responsibility now, so that when I'm that age, the change is there. That I don't have the same problem later on."(V-F2B5)<br><br>"Every time I go out there (...) so that's bad. And many of them just don't leave it any more, not at all. And this realization sometimes gets to me."(V-F2B2)	Vs' self-perceived psychological overload due to intervention	Overloaded 0 (0)<br>More likely overloaded 1 (2.5)<br>Neither nor 1 (2.5)<br>More likely not overloaded 11 (27.5)<br>Not overloaded 27 (67.5)
