# Supplementary material for: Experiences of participants of a volunteer-supported walking intervention to improve physical function of nursing home residents – a mixed methods sub-study of the POWER-project
Source: BMC Geriatr. 2023 Jun 1;23:343. doi: 10.1186/s12877-023-04044-4 (PMC10234228; doi:10.1186/s12877-023-04044-4)
Supplement: Supplementary file 5 — Supplementary Material 5 [file 12877_2023_4044_MOESM5_ESM.pdf]

Additional file 3. Additional experiences of intervention

*NHR=nursing home resident, V=Volunteer, 1=individual interview, 2=focus group, *multiple response possible, †=data given for 54 NHR*

	Qualitative study		Quantitative Study	
	**Key results**	**Example**	**Variables assessed in the questionnaire**	**Response category N (%)**
**Behavior of NHR[2]**	Consensus:<br>+ motivation for walks and exercise<br>+ Need to respond to NHR<br>V:<br>- Motivation depends on daily form/health	"The motivation was always very different. It is always according to the personal situation. Whether the lady feels good that day or not. Sometimes there was also a day when I thought, why doesn't she want to go out now?" (V-F2B5)<br><br>"I did not have the problem that it he was not motivated. It was basically very very nice. He really wanted to do it." (V-F2B2)	Cancelled appointments by NHR*†	Health conditions NHR 24 (44.4)<br>Motivation NHR 14 (25.9)<br>Weather 8 (14.8)<br>Other 8 (14.8)<br>No 14 (25.9)<br>Don't know 0 (0)<br>Not stated 0 (0)<br>Missing 0 (0)
**Behavior of Volunteers[1]**	Consensus:<br>+ motivation for walks and exercise<br>+ Need to respond to NHR<br>NHR:<br>-Few volunteers were less qualified for support, unreliable or dropped out of the project without informing the NHR.	"And I now walk with the walker and I think that's so nice because she's still a bit younger. She always takes the steps the way I do. And I don't have to say, Ms XX not so fast, I can't do that. So she adapts to me." (NHR-W9)<br>" …we always got along and talked quite well. I don't know why she broke it off. She had somehow fallen ill and were on holiday and so on." (NHR-W6)	n/a	n/a
**protection/safety for NHR on walks [1,2]**	Consensus:<br>+ Accompaniment takes away fear of falling, gives safety and protection	"Yes, but I mean, I can also go for a walk alone. Nevertheless, the danger is there/ Then you trip, fall and then you are alone and then you are standing there. And that's not really possible"(NHR-W6)<br><br>"Of course she wouldn't have done it on her own, she would have been too scared or something, but this way she had the confidence to do it voluntarily. And then on the way back she also went up the stairs again." (V-F1B7)	n/a	n/a

Additional file 3. Additional experiences of intervention
*NHR=nursing home resident, V=Volunteer, 1=individual interview, 2=focus group, *multiple response possible, †=data given for 54 NHR*

	Qualitative study		Quantitative Study	
	**Key results**	**Example**	**Variables assessed in the questionnaire**	**Response category N (%)**
**Relationship of walking partners[1,2]**	<u>Consensus</u>:<br>+ Almost without exception, good to very good friendly relationship<br>+ Often trusting relationship (issuing powers of attorney etc.)<br><u>NHR:</u><br>Some NHRs allowed only a limited amount of closeness and kept distance.	"Ms XX, that could be my sister" (NHR-W9)<br><br>"A friendly relationship, yes. But a relationship of trust, no. I haven't tried that out yet either. What should I trust her with? With my finances? I always think that's none of the other person's business." (NHR-W9)	Relationship to NHR[†]	Very good 25 (46.3)<br>Good 22 (40.7)<br>Moderate 2 (3.7)<br>Bad 1 (1.9)<br>Don't know 4 (7.4)<br>Not stated 0 (0)<br>Missing 0 (0)
		"…and that this very personal with her sister was discussed, now that was out of the situations, but it shows me that she does have a certain amount of trust." (V-F1B2)<br><br>"She said: The good Lord sent you"(...) I even have power of attorney. So that means something, doesn't it? (...) When you have such confidence that you are allowed to do this and that, that is unbelievable."(V-F1B4)	Developing a relationship of trust with the NHR[†]	Yes 26 (48.1)<br>Most likely yes 14 (25.9)<br>Most likely no 8 (14.8)<br>No 3 (5.6)<br>Don't know 3 (5.6)<br>Not Stated 0 (0)<br>Missing 0 (0)
