# Supplementary material for: Experiences of participants of a volunteer-supported walking intervention to improve physical function of nursing home residents – a mixed methods sub-study of the POWER-project
Source: BMC Geriatr. 2023 Jun 1;23:343. doi: 10.1186/s12877-023-04044-4 (PMC10234228; doi:10.1186/s12877-023-04044-4)
Supplement: Supplementary file 6 — Supplementary Material 6 [file 12877_2023_4044_MOESM6_ESM.pdf]

Additional file 6. Awareness of project, training & support
*NHR=nursing home resident, V=Volunteer, 1=individual interview, 2=focus group, *multiple response possible, †=data given for 54 NHR*

	Qualitative study		Quantitative Study	
	**Key results**	**Example**	**Variables assessed in the questionnaire**	**Response category n (%)**
**Project attention**	n/a	n/a	**Project attention***	Press/Radio 22 (55.0)<br>Friends 8 (20.0)<br>Flyer 7 (17.5)<br>Volunteer agency 2 (5.0)<br>Nursing home 1 (2.5)<br>Other 4 (10.0)<br>Don`t know 0 (0)<br>Not stated 0 (0)<br>Missing 0 (0)
**Experience with elderly before project start**	n/a	n/a	**Experience with elderly before project start***	Care in private sector 19 (47.5)<br>Professional experience 8 (20.0)<br>Voluntary activities 8 (20.0)<br>No 12 (30.0)<br>Don`t know 0 (0)<br>Not stated 0 (0)<br>Missing 0 (0)
**Important topics included in training**	n/a	n/a	**Important topics included in training**	Yes 33 (82.5)<br>More likely yes 6 (15.0)<br>More likely no 0 (0)<br>No 0 (0)<br>Don't know 1 (2.5)<br>Not stated 0 (0)<br>Missing 0 (0)
**Helpfulness of training material**	n/a	n/a	**Helpfulness of training material**	Yes 23 (57.5)<br>More likely yes 10 (25.0)<br>More likely no 7 (17.5)<br>No 0 (0)<br>Don't know 0 (0)<br>Not stated 0 (0)<br>Missing 0 (0)
**Participation in experience change meeting**	n/a	n/a	**Participation in experience change meeting**	Yes 31 (77.5)<br>No 8 (20)<br>Not stated 0 (0)<br>Missing 1 (2.5)
