# Supplementary material for: Experiences of participants of a volunteer-supported walking intervention to improve physical function of nursing home residents – a mixed methods sub-study of the POWER-project
Source: BMC Geriatr. 2023 Jun 1;23:343. doi: 10.1186/s12877-023-04044-4 (PMC10234228; doi:10.1186/s12877-023-04044-4)
Supplement: Supplementary file 7 — Supplementary Material 7 [file 12877_2023_4044_MOESM7_ESM.pdf]

Additional file 5. Project organization
*NHR=nursing home resident, V=Volunteer, 1=individual interview, 2=focus group, *multiple response possible, †=data given for 54 NHR*

	Qualitative study		Quantitative Study	
	**Key results**	**Example**	**Variables assessed in the questionnaire**	**Response category N (%)**
**Matching NHR/VI$_{1,2}$**	NHR:<br>+ Differences age/gender rather positive<br>V:<br>+ random assignment no disadvantage<br>- Test persons sometimes not sufficiently informed about the project<br>- Accompaniment of socially involved persons (family, friends) less meaningful	“It is not a question of age “ (NHR-W8)<br><br>“That's why I didn't take a woman now. Because I always have to talk. I can't have that. Then when I want to talk, I talk and don't let others cut me off.”(NHR-W4)<br><br>“…the person we both had together, she has a lot of people who visit her and then go for walks with her.” (V-F1B1)<br><br>“If it was someone who was totally single and had no one to go with them, then it would make sense to accompany them.” (V-F1B7)	n/a	n/a
**V-selection/recommendations for improvement$_1$**	NHR:<br>+ higher allowance for volunteer work.<br>+ motivated, reliable Vs preferred	“To choose the partner. That is important. And they should perhaps, sure, money is tight everywhere, but they should perhaps be compensated a bit more. So that the motivation is there and the pocket money is increased.”(NHR-W8)	Further voluntary engagement conceivable	Yes 28 (70.0)<br>More likely yes 5 (12.5)<br>More likely no 3 (7.5)<br>No 0 (0)<br>Don't know 3 (7.5)<br>Not stated 1 (2.5)<br>Missing 0 (0)
			Important requirements for voluntary work*	Accessibility of place of work 33 (84.6)<br>Time flexibility 29 (74.4)<br>Insurance for V 23 (59.0)<br>Professional support 21 (53.8)<br>Certificates etc. 10 (25.6)<br>Financial compensation 2 (5.1)<br>Don’t know 1 (2.6)<br>Not stated 0 (0)<br>Missing 0 (0)

Additional file 5. Project organization
*NHR=nursing home resident, V=Volunteer, 1=individual interview, 2=focus group, *multiple response possible, †=data given for 54 NHR*

	Qualitative study		Quantitative Study	
	**Key results**	**Example**	**Variables assessed in the questionnaire**	**Response category N (%)**
**NHR-selection/recommendations for improvement[1,2]**	Consensus:<br>+ Mainly involve socially isolated people in project:<br>NHR:<br>Project more suitable for younger/less physically ill people.<br>V:<br>+ involve family and friends of NHR in programme.<br>+ form teams of test persons/alliances in the nursing home<br>+ Programme needed for people living alone in need of assistance<br>- Constant encouragement by V necessary to continue the programme	"Those who are younger than me would perhaps do better (...). But I'm telling you, most of the people who are here are all in wheelchairs " (NHR-W1)<br><br>"…and I can't imagine that (...) it will be continued independently on its own. It quickly goes into the old rut, if one had perhaps forged alliances, in pairs, small groups, not groups, two, then perhaps one would have pulled the other along" (V-F1B2)<br><br>"I sometimes think that someone who is at home and turns on the TV in the morning, or then the care service comes at seven o'clock and at ten o'clock the food is put in front of the door and otherwise he has no one all day, that I would rather look for such people privately or from something, from some organization, because I think it is even more necessary there" (V-F1B5).	n/a	n/a
