# Supplementary material for: Experiences of participants of a volunteer-supported walking intervention to improve physical function of nursing home residents – a mixed methods sub-study of the POWER-project
Source: BMC Geriatr. 2023 Jun 1;23:343. doi: 10.1186/s12877-023-04044-4 (PMC10234228; doi:10.1186/s12877-023-04044-4)
Supplement: Supplementary file 8 — Supplementary Material 8 [file 12877_2023_4044_MOESM8_ESM.pdf]

Additional file 4. Challenges of intervention for NHR and V
*NHR=nursing home resident, V=Volunteer, 1=individual interview, 2=focus group, *multiple response possible, †=data given for 54 NHR*

	Qualitative study		Quantitative Study	
	**Key results**	**Example**	**Variables assessed in the questionnaire**	**Response category N (%)**
**Responsibility for NHR's$_2$**	+ Safety due to proximity to the nursing home/contact person	"…we actually had a very great safety, the nursing home was close by, if anything happened, that made the whole work very easy for us" (V-F1B5).	Support from nursing home/contact person†	Yes 32 (59.3)<br>No 21 (38.9)<br>Don't know 0 (0)<br>Not stated 0 (0)<br>Missing 1 (1.9)
**Challenges due to the NHR's behaviour$_2$**	- Changing motivation to walks and exercise<br>- Existing physical and cognitive diseases and limitations<br>- In some cases, significant persuasion work is required	"He was also very forgetful, so much that when I asked him where we were, where we had been before and so on, he couldn't remember at all." (V-F1B1)<br><br>"Because she often doesn't want to. You can't force her. I try to motivate her. But that doesn't always work. She has days when she goes, but then again she doesn't" (V-F2B1).	Cancelled appointments by NHR*†	Health conditions NHR 24 (44.4)<br>Motivation NHR 14 (25.9)<br>Weather 8 (14.8)<br>Other 8 (14.8)<br>Don't know 0 (0)<br>No 14 (25.9)<br>Not stated 0 (0)<br>Missing 0 (0)
**Uncertainty/overload $_{1,2}$**	<u>NHR</u>:<br>- Physical overload due to walks<br><u>V:</u><br>- Adverse events unsettled V<br>+ no interruption/discontinuation of walks due to Adverse events	"When it was over, I was happy. Then I was so exhausted that the first thing I did was sit down on the bed or the armchair." (NHR-W5)<br><br>"I was (...) was pretty much at the beginning, still a stranger, not doing anything big (…) and she can't go any further. Oh, I ran to bring the wheelchair down. When she was sitting in it, I was happy. I brought her up, water, water, water, and then I went to the nursing staff and told them, but as I said: in retrospect it was nothing bad, but in the situation: no, it wasn't great."(V-F1B2)	V's self-perceived physical overload due to intervention<br><br><br><br><br><br><br><br>Vs' self-perceived psychological overload due to intervention	More likely Overloaded 0 (0)<br>More likely overloaded 1 (2.5)<br>More likely not overloaded 4(10.0)<br>Not overloaded 35 (87.5)<br>Don't kow 0 (0)<br>Not stated 0 (09<br>Missing 0 (0)<br><br>Overloaded 0 (0)<br>More likely overloaded 1 (2.5)<br>More likely not overloaded 11 (27.5)<br>Not overloaded 27 (67.5)<br>Don't know 1 (2.5)<br>Not stated 0 (0)<br>Missing 0 (0)

Additional file 4. Challenges of intervention for NHR and V
*NHR=nursing home resident, V=Volunteer, 1=individual interview, 2=focus group, *multiple response possible, †=data given for 54 NHR*

	Qualitative study		Quantitative Study	
	**Key results**	**Example**	**Variables assessed in the questionnaire**	**Response category N (%)**
**Discontinuation of intervention by NHR1,2**	V:<br>- Lack of information about project/intervention led to discontinuations	"I was glad when that was over. Because then I was no longer able to cope so well. Therefore, I was glad when it was over. Yes, I was then also sick and then somehow it was bad once. Now I am lying down again. That time is also over and it's also quite good that it's over now."(NHR-W7)<br><br>"The second lady didn't really know herself what she was getting into, I think. And after a short time she said, "no, I don't need to take part in any more Olympics"."(V-F1B2)	Discontinuation of intervention by NHR (observational or extended period)*†	Health conditions 2 (3.7)<br>Lack of motivation 10 (18.5)<br>Lack of time 0 (0)<br>Other(e.g. end of observational period) 16 (29,6)<br>No 21 (38.9)<br>Don't know 1 (1.9)<br>Not stated 4 (7.4)<br>Missing 0(0)
**Challenges due to general conditions1,2**	Consensus:<br>- Surroundings of nursing home inappropriate for walks.<br>- Weather conditions<br>NHR:<br>- fixed appointment stressed	"…because this home, so it's just it's steep uphill and downhill…"(V-F1B1)<br><br>"As I said, that it's so bumpy here. Yes, the pavement, the stones are also broken and when you're with the walker, it's all a bit bumpy." (NHR-W3)<br><br>"Yes, there was a certain time, now you have to be ready. You have to be ready now. Do you have to go to the toilet first or not? I can't do that well anymore. That's all that doesn't work anymore." (NHR-W1)	Surroundings suitable for walks*	Yes 33 (61.1)<br>No 19 (35.2)<br>Don't know 0 (0)<br>Not stated 1 (1.9)<br>Missing 1 (1.9)
