# Supplementary material for: Experiences of participants of a volunteer-supported walking intervention to improve physical function of nursing home residents – a mixed methods sub-study of the POWER-project
Source: BMC Geriatr. 2023 Jun 1;23:343. doi: 10.1186/s12877-023-04044-4 (PMC10234228; doi:10.1186/s12877-023-04044-4)
Supplement: Supplementary file 9 — Supplementary Material 9 [file 12877_2023_4044_MOESM9_ESM.pdf]

Questionnaire to evaluate the experience of
# of volunteer walking companions in the POWER Study
(A volunteer-supported walking programme to improve physical function in older people)

Please answer the following questions:

1. How did you find out about the POWER project?
(multiple answers possible)

☐ friends ☐ flyer

☐ volunteer agency ☐ nursing home

☐ press/TV/radio

☐ other: ____

☐ don't know ☐ n/a

2. Before the project, did you already have experience in dealing with older people or nursing home residents?
(multiple answers possible)

☐ yes, by caring for relatives/friends/neighbours

☐ yes, professional experience as ____

☐ yes, through previous voluntary work

☐ no

☐ don`t know

☐ n/a

3. What motivated you to sign up for the POWER project?

(multiple answers possible)

☐ to receive recognition	☐ to make new contacts
☐ Interest in exercise	☐ Interest in scientific study
☐ Meaning of life	☐ to gain new experiences
☐ Improvement of own health	☐ Financial compensation (expense allowance)
☐ other: ____________________	
☐ don't know	☐ n/a

4. Did the training address all the topics that are important to you?

☐ strongly agree	☐ agree
☐ strongly disagree	☐ disagree
☐ don't know	☐ n/a

I missed the following topics: ____________________

5. Was the training manual helpful to you during the period of regular walks?

☐ yes	☐ rather yes
☐ no	☐ rather no
☐ don't know	☐ n/a

6. Have you participated in the meetings for the exchange of experience?

☐ yes

☐ no, because: ____

☐ n/a

7. How often did you go for a walk with your walking companion during the week?

☐ less than 1x/week ☐ 1x/week

☐ 2x/week ☐ 3x/week

☐ more than 3x/week ☐ don’t know

☐ n/a

8. Was the environment around your walk companion's nursing home suitable for walks with seniors?

☐ yes

☐ no, because: ____

☐ don’t know ☐ n/a

9. Have you been or will you be adequately supervised and supported by the contact person/caregiver in your volunteer role?

☐ yes

☐ no, because: ____

☐ don’t know ☐ n/a

10. How was the relationship between you and your walking partner?

☐ very good ☐ good

☐ less good ☐ bad

☐ don’t know ☐ n/a

11. Has a relationship of trust been established with your walking partner?

☐ yes ☐ rather yes

☐ no ☐ rather no

☐ don’t know ☐ n/a

12. Has your physical performance and health changed as a result of the walks and activities?

☐ improved ☐ rather improved

☐ worse ☐ rather worse

☐ same

☐ don’t know ☐ n/a

13. Have you been physically stressed by the regular company of your walking partner?

☐ yes ☐ rather yes

☐ no ☐ rather no

☐ don’t know ☐ n/a

14. Have you been psychologically burdened by the regular companionship of your walking partner?

☐ yes ☐ rather yes

☐ no ☐ rather no

☐ don’t know ☐ n/a

15. Have appointments been cancelled by your walking partner?

(multiple answers possible)

☐ yes, because:

- ☐ weather
- ☐ illness of the walk partner
- ☐ motivation of the walk partner
- ☐ other reasons:____

☐ no

☐ don’t know

☐ n/a

16. Was the project cancelled by your walk partner before the start of the corona contact lockout?

(multiple answers possible)

☐ yes, because :

➔ **continue with question 20**

- ☐ bad health condition
- ☐ too little time
- ☐ lack of interest
- ☐ other reasons:

____

☐ no

☐ don’t know

☐ n/a

With the outbreak of the Corona pandemic (COVID19/SARS-CoV-2) in Germany, visits to nursing homes and homes for the elderly were forbidden by the government of North Rhine-Westphalia on 13.03.2020:

17. At the beginning of the visitation ban, did you still have contact to your walking partner?

☐ yes, in fact

☐ walks

☐ visits

☐ other ____

☐ no ➔ **continue with question 20**

18. Were you able to keep in contact with your walking partner during the visitation ban?

☐ yes, in fact

☐ walks/visits

☐ telephone calls

☐ correspondence (postcard/letter/WhatsApp/SMS)

☐ other: ____

☐ no

19. Will you continue the walks with your walking partner after the visitation ban ends?

☐ yes

☐ rather yes

☐ no

☐ rather no

☐ don’t know

☐ n/a

20. Will you continue your volunteer engagement in the future?

☐ yes ☐ rather yes

☐ no ☐ rather no

☐ don’t know ☐ n/a

21. Which of the items below are important to you when volunteering:

(multiple answers possible)

☐ Liability and accident insurance provided by the organization

☐ Recognition of the activity in the form of certificates, identification, honours or similar.

☐ professional support

☐ financial remuneration/expense allowance

☐ good accessibility of the place of use

☐ time flexibility

☐ don’t know

☐ n/a

22. Is there anything else you would like to tell us?

Please share your suggestions, wishes, comments or criticism with us:

**Personal information:**
☐ female ☐ male
Age in years: ___________________
Number of walking partners in total: _____________________

Please return the questionnaire in the enclosed stamped envelope to:

You are also welcome to fax back the questionnaire and your consent form or send it by e-mail to:

Fax-No.: 02302 926 745

E-Mail: power@uni-wh.de

Please return ALL pages of the questionnaire as well as your signed consent form (in a separate envelope or separately), too.

Many thanks for your support!
